# Supplementary material for: Lipid Nanoparticles as a Shuttle for Anti-Adipogenic miRNAs to Human Adipocytes
Source: Pharmaceutics. 2023 Jul 19;15(7):1983. doi: 10.3390/pharmaceutics15071983 (PMC10384627; doi:10.3390/pharmaceutics15071983)
Supplement: Supplementary file 1 [file pharmaceutics-15-01983-s001.zip › pharmaceutics-2480025-supplementary.pdf]

Supplementary Material

Lipid Nanoparticles as a Shuttle for Anti-Adipogenic miRNAs to Human Adipocytes

Anna-Laurence Schachner-Nedherer [1,2,†], Julia Fuchs [1,†], Ivan Vidakovic [1], Oliver Höller [1], Gebhard Schratter [1], Gunter Almer [3], Eleonore Fröhlich [4], Andreas Zimmer [2], Martin Wabitsch [5], Karin Kornmueller [1] and Ruth Prassl [1,*]

[1] Gottfried Schatz Research Center for Cell Signaling, Metabolism and Aging, Division of Medical Physics and Biophysics, Medical University of Graz, 8010 Graz, Austria; anna.schachner-nedherer@medunigraz.at (A.-L.S.-N.); julia.fuchs@medunigraz.at (J.F.); ivan.vidakovic@medunigraz.at (I.V.); oliver.hoeller@medunigraz.at (O.H.); karin.kornmueller@medunigraz.at (K.K.)

[2] Department of Pharmaceutical Technology and Biopharmacy, Institute of Pharmaceutical Sciences, University of Graz, 8010 Graz, Austria; andreas.zimmer@uni-graz.at

[3] Clinical Institute for Medical and Chemical Laboratory Diagnostics, Medical University of Graz, 8010 Graz, Austria; gunter.almer@medunigraz.at

[4] Center for Medical Research, Medical University of Graz, 8010 Graz, Austria; eleonore.froehlich@medunigraz.at

[5] Division of Pediatric Endocrinology, Diabetes Department of Pediatrics and Adolescent Medicine, University Medical Center Ulm, 89075 Ulm, Germany; martin.wabitsch@uniklinik-ulm.de

* Correspondence: ruth.prassl@medunigraz.at; Tel.: +43-316-385-71695

† These authors contributed equally to this work.

Supplementary data

Table S1. *RT-qPCR primer sequences.*

Organism	Gene symbol	RefSeq ID	Forward primer (5′-3′)	Reverse primer (5′-3′)
hsa	GAPDH	NM_005276	TTGTGGTGCCCCATCAGTTC	CCCAATCACTTCCGAGATGA
hsa	PPARγ	NM_138712	AGCCTCATGAAGAGCCTTCCA	TCCGGAAGAAACCCTTGCA
hsa	UCP1	NM_021833	GTGTGCCCAACTGTGCAATG	CCAGGATCCAAGTCGCAAGA

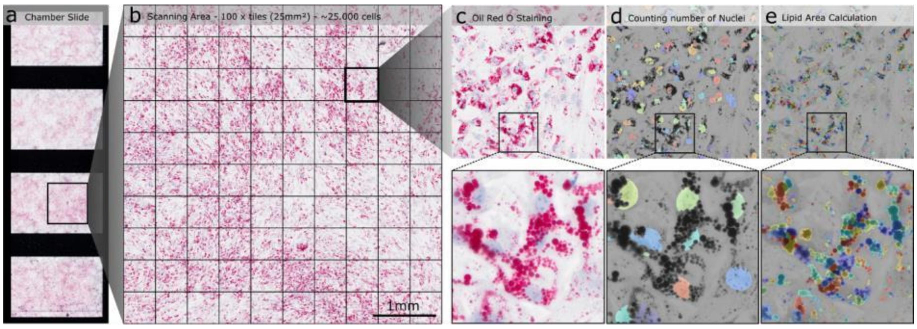

Figure S1. Schematic illustration of automated quantitative image analysis of ORO staining. Olympus SLIDEVIEW VS200 slide scanner was used to scan an entire 4-well chamber slide in brightfield (20x) as shown in (a). Two chambers are always under the same treatment conditions. (b) A 25 mm^2 area (~25 000 cells) of each chamber was used and split into 100 non-overlapping single images for automated quantitative image analysis using CellProfiler software. (c) shows a single image of OROstained overview after splitting. (d) depicts the same image as in (c) as an overlay image of lipid droplet areas (greyscale) with detected cell nuclei (colored regions). Nuclei were counted for each image. (e) depicts the automated detection of lipid droplets (colored areas) of quantitative image analysis on the greyscale image of (c). Black boxes in figures (c), (d) and (e) indicate the areas that are displayed at higher magnification below. The lipid area of each image was calculated and divided by the number of nuclei corresponding to the image. The analysis (a–e) was conducted for each pretreatment. Results are shown in Figure 5.

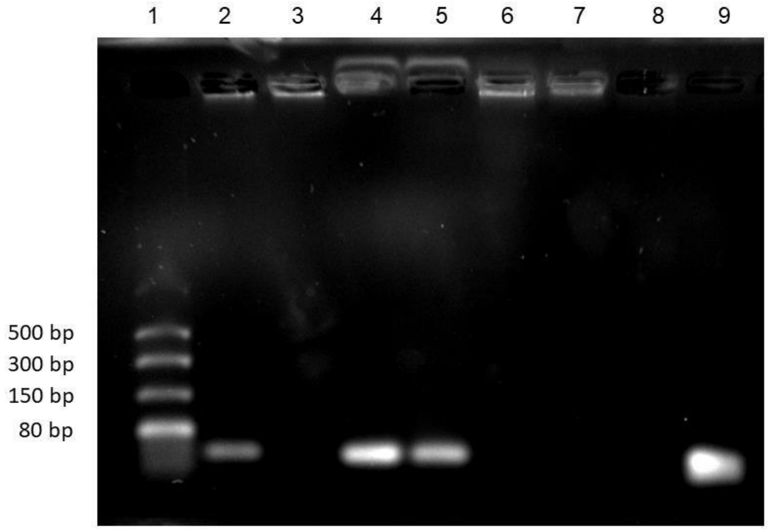

Figure S2. Stability of PEG-LNP/miRNA complexes after 3 weeks storage at 4°C. Same samples as shown in Figure 1B. When dialysed and stored in PBS buffer pH:7.5 about 60% of the miR26a was released (lane 2), which was accessible to nuclease digestion (lane 6). Complexed miR26a was finally released upon addition of Triton X-100 (lane 4). No release of miRNA could be detected when dialysed against PBS buffer pH:5.5 and stored for 3 weeks (lane 3). Further, the miRNA was shielded from nuclease digestion (lane 7) but could be released upon particle disruption with Triton X-100 (lane 5). Free miRNA (lane 9) could be readily digested by addition of nuclease (lane 8). Marker (lane1).

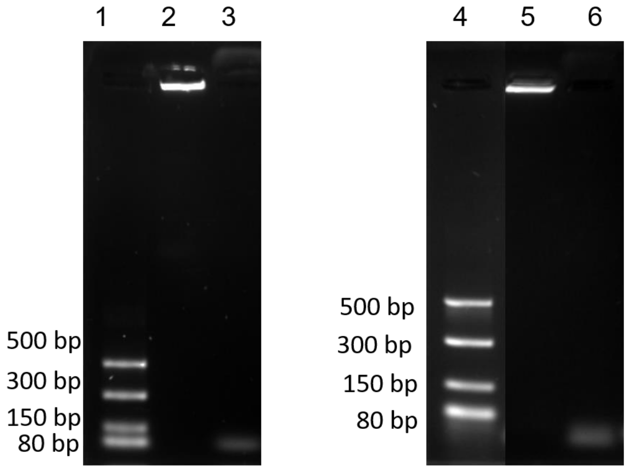

Figure S3. Complexation efficiency and stability of HiPerFect/miR26a after batch production (left panels) and after 7 days storage at 4◎C (right panels). HiPerFect mixed with miR26a (lane 2) and after treatment with Triton X-100 (lane 3). Note, the applied concentration of miRNA in the Triton X-100 treated sample is three times less than in the HiPerFect complexed sample (lane 2). HiPerFect/miR26a after storage (lane5) and after Triton X-100 treatment (lane 6). Marker (lane 1 and lane 4).

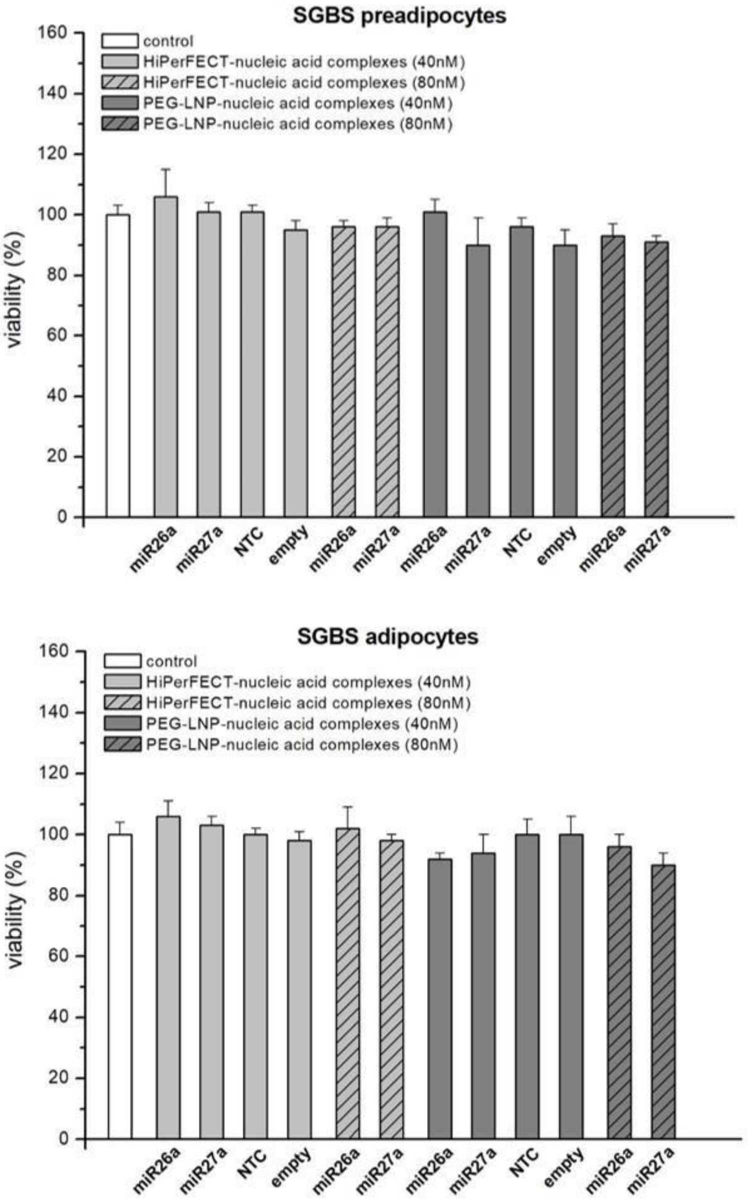

Figure S4. Cell viability after 24h incubation of PEG-LNP (dark grey bars) and HiPerFECT (grey bars) containing 40nM (plain grey and dark grey bars) and 80nM (dashed grey and dark grey bars) miR26a, miR27a, NTC or unfilled (empty). The particles were tested on SGBS preadipocytes (A) and SGBS adipocytes at d8 (B).
